# Suhexiang pill for acute ischemic stroke in real-world practice setting (SUNRISE): protocol of a multicenter registry

**DOI:** 10.1186/s12906-025-04762-9

**Published:** 2025-01-28

**Authors:** Xinxing Lai, Xuejiao Xiong, Qi Jia, Tingting Liu, Zhaowen Yang, Chi Zhang, Lingbo Kong, Kegang Cao, Ting Dong, Caixia Fang, Jianwen Ge, Li Dong, Zhitao Zong, Sisi Chen, Yuhong Ma, Xue Bai, Dahua Wu, Yao Xie, Mingyan Zhang, Yilong Wang, Guohui Jiang, Daqiao Song, Yanping Wang, Chunyan Gui, Qingwen Geng, Ying Gao, for the SUNRISE study investigators

**Affiliations:** 1 Department of Neurology, Dongzhimen Hospital, Beijing University of Chinese Medicine, Beijing, China; 2 Institute for Brain Disorders, Beijing University of Chinese Medicine, Beijing, China; 3 Beijing University of Chinese Medicine, Beijing, China; 4 Department of Neurology, The Affiliated Hospital of Shandong University of Traditional Chinese Medicine, Jinan, China; 5 Department of Neurology, The First Affiliated Hospital of Anhui University of Traditional Chinese Medicine, Hefei, China; 6 Department of Pharmacy, Qingyang People’s Hospital, Qingyang, China; 7 Department of Neurology, Tianshui Hospital of Traditional Chinese Medicine, Tianshui, China; 8 Department of Neurosurgery, Jiujiang First People’s Hospital, Jiujiang, China; 9 Department of Acupuncture and Moxibustion, Jiujiang Hospital of Traditional Chinese Medicine, Jiujiang, China; 10 Department of Neurology, The Affiliated Hospital of Changzhi Institute of Traditional Chinese Medicine, Changzhi, China; 11 Department of Neurology, The Affiliated Traditional Chinese Medicine Hospital of Southwest Medical University, Luzhou, China; 12 Department of Neurology, Hunan Integrated Traditional Chinese and Western Medicine Hospital, Changsha, China; 13 Department of Neurology, Shehong People’s Hospital, Shehong, China; 14 Intensive Care Unit, Shehong People’s Hospital, Shehong, China; 15 Department of Neurology, The Affiliated Hospital of North Sichuan Medical College, Nanchong, China; 16 Department of Neurology, Yiwu Hospital of Traditional Chinese Medicine, Yiwu, China; 17 Department of Neurology, Jiaxing Second Hospital, Jiaxing, China; 18 Department of Neurology, Dazhou Central Hospital, Dazhou, China; 19 Chinese Medicine Key Research Room of Brain Disorders Syndrome and Treatment of the National Administration of Traditional Chinese Medicine, Beijing, China

**Keywords:** Acute ischemic stroke, Suhexiang pill, Traditional Chinese medicine, Real world, Registry

## Abstract

**Background:**

Suhexiang (SHX) pill is widely used for treating acute ischemic stroke (AIS). Experimental and randomized controlled trials suggested that SHX pill was beneficial for patients with AIS. However, the effectiveness of SHX pill in real-world practice setting remains unclear. It is of great importance to investigate the effectiveness and safety of SHX pill in patients with acute ischemic stroke in real-world clinical practice with long-term follow-up.

**Methods:**

The Suhexiang pill for acute ischemic stroke in Real-world Practice Setting (SUNRISE) is a multicenter, prospective, product-specific, observational study designed to provide insight into the administration of SHX pill for patients with AIS in the real-world clinical practice setting, with an initial sample size of 1000. Eligible patients treated with SHX pill within seven days of AIS onset will be consecutively included in this registry. The primary outcome is the proportion of patients independent at 3 months after stroke onset defined by an mRS score of 0, 1, or 2.

**Conclusion:**

The findings of the SUNRISE registry will not only provide insights into the characteristics of patients who may benefit from SHX treatment, but also may enable the individualized treatment decision-making of SHX pill in real-world practice setting.

**Study registration:**

This study was registered with the ClinicalTrials.gov (URL: https://clinicaltrials.gov/, Unique identifier: NCT05833932).

**Supplementary Information:**

The online version contains supplementary material available at 10.1186/s12906-025-04762-9.

## Introduction

Stroke is one of the leading causes of death and long-term disability in adults worldwide [1, 2]. Globally, the estimated lifetime risk of stroke over the next 25 years is highest in China [3]. A recent cross-sectional study based on a nationally representative survey reported that in 2020, the estimated overall prevalence, incidence, and mortality rate of stroke were 2.6%, 505.2 per 100 000 person-years, and 343.4 per 100 000 person-years, respectively, which indicated approximate 17.8 million cases of stroke, 3.4 million new strokes, and 2.3 million stroke-related deaths in China [4]. A significant financial burden results from stroke-related treatment, post-stroke rehabilitation, and care. It has been reported that China bears the highest economic cost related to stroke globally [5]. The majority of strokes are ischemic due to reduced or blocked blood flow, generally resulting from arterial occlusion, which accounts for 70–83% of all stroke cases in China [6].

Despite substantial advances in reperfusion therapy for acute ischemic stroke during the past decades, only a minority of highly selected patients could receive thrombolytic therapy due to strict criteria. A cross-sectional study of 938 tertiary hospitals and 786 secondary hospitals from 31 provinces in China reported that the overall intravenous thrombolysis for AIS was 5.64%, and the endovascular therapy rate was 1.45%, respectively, indicating that the majority of patients with AIS were not eligible for reperfusion treatments [7]. For those patients who have not received reperfusion therapy, the lack of effective and widely applicable or accessible therapeutic treatments for AIS has resulted in an increasing interest in traditional Chinese medicine (TCM) [8–10].

Suhexiang (SHX) pill is a well-known Chinese patent medicine, mainly manufactured by Lei Yun Shang Pharmaceutical Group Co., Ltd, which has been approved by the National Medical Products Administration of China (approval number: Z32020480) for the treatment of stroke, seizures, infantile convulsion, sudden loss of consciousness, and other dysfunctions [11]. Experimental studies reported that SHX could significantly improve long-term neurological outcomes in the focal stroke model of rats and exert the inhibition of acute proinflammation activation [12]. Meanwhile, SHX pill exerts antiepileptic and anticonvulsant effects, modulating neurotransmitter imbalance and suppressing the central nervous system by increasing the inhibitory neurotransmitter γ-aminobutyric acid (GABA) and inhibiting the excitatory neurotransmitter glutamate [13–15]. In addition, SHX pill confers a therapeutic potential to AD-like pathology of Aβ42 overexpressing Drosophila model via suppression of the hyperactivation of JNK activity and apoptosis, as well as a potential as a therapeutic drug for the treatment of AD and Parkinson’s disease [16–18]. Moreover, several randomized controlled trials (RCTs) suggested that SHX pill combined with conventional treatments showed beneficial effects on neurological function compared with conventional therapy alone [19, 20]. For instance, Yang et al. reported a randomized controlled trial with 98 patients of acute ischemic stroke, in which SHX combined with clopidogrel produced significant improvement of the National Institute of Health Stroke Scale (NIHSS) compared with clopidogrel alone (6.79 ± 2.01 vs. 4.66 ± 1.73, *P* < 0.05) [19].

Despite the promising findings from experimental and clinical trials, the effectiveness of SHX pill in real-world practice setting remains unclear, which has limited its optimal usage in routine practice. It is of great importance to investigate the epidemiology and clinical characteristics of patients who received SHX treatments. Because the RCTs only included highly selected patients with AIS and excluded special populations such as elderly patients, critically ill patients or patients with complicated comorbidities, resulting in little evidence regarding the effectiveness and safety of SHX in these populations. The generalizability of findings from RCTs remains unknown, therefore, these results should be interpreted with caution before more data from real-world clinical practice are available.

The primary objective of the Suhexiang pill for acute ischemic stroke in real-world practice setting (SUNRISE) registry is to evaluate the effectiveness and safety of SHX pill in patients with acute ischemic stroke in routine clinical practice with a one-year follow-up.

## Methods and design

### Study design

The SUNERISE registry is a multicenter, prospective, product-specific, observational study designed to provide insight into the administration of SHX pill for patients with AIS in the real-world clinical practice setting. Physicians decide whether to prescribe SHX pill based on their assessment of the patient’s overall condition, the drug’s stated indications, clinical guidelines, and the patient’s preferences. This present registry is one of the cohorts in the China Stroke Registry for Patients with Traditional Chinese Medicine (CASES-TCM), which is a prospective, multicenter, observational disease-specific registry [21]. Recruitment began on May 1, 2023, and the registry is scheduled to complete recruitment of all human participants by December 31, 2024. The SUNRISE registry has been approved by the Institutional Review Board of Dongzhimen Hospital, Beijing University of Chinese Medicine, Beijing, China (No. 2022DZMEC-268-02), as well as the local ethical committees of all participating sites.

### Study population

Patients will be included in the SUNRISE registry if they meet all the inclusion and none of the exclusion criteria:

#### Inclusion criteria

- All adult patients (≥ 18 years of age) admitted to the participating hospitals with a diagnosis of acute ischemic stroke.
- Acute ischemic stroke within seven days of symptom onset.
- Treated with Suhexiang pill.
- Patient or legally authorized representative has signed written informed consent.

#### Exclusion criteria

- Known to be allergic to Suhexiang pill or related ingredients.
- Comorbidities such as severe cognitive impairment, aphasia, or osteoarthritis that may significantly affect the outcomes assessment including neurological function and activities of daily living.
- Known to be pregnant or breastfeeding.

Importantly, patients are still eligible to participate in this registry if they were previously treated with SHX pill for another condition before the stroke.

### Treatments

SHX pill contains fifteen Chinese herbal medicines [14]: *Styrax* (Su Hexiang), *Benzoinum* (An Xixiang), *Borneolum Syntheticum* (Bing Pian), *Cornu Bubali* (Shui Niujiao), *Moschus* (She Xiang), *Santalum album Linn.* (Tan Xiang), *Aquilariae Lignum Resinatum* (Chen Xiang), *Syzygium aromaticum* (Ding Xiang), *Cyperi Rhizoma* (Xiang Fu), *Radix Aucklandiae* (Mu Xiang), *Boswellia sacra* (Ru Xiang), *Piper longum Linn.* (Bi Ba), *Rhizoma Atractylodis Macrocephatae* (Bai Zhu), *Frctus Terminaliae Chebulae* (He Zirou) and *Cinnabaris* (Zhu Sha). SHX is recommended to be taken orally, with the dosage of one or two pills, one or twice a day, for at least seven days. However, the actual dosage and treatment course of SHX pill will be determined at the discretion of the treating physician as well as the discussion between the patients and the physician. In addition, all participants are allowed to receive routine stroke care in accordance with the guidelines [22], including (but not limited to) intravenous thrombolysis, endovascular treatment, antiplatelet, blood pressure control, as well as management of other risk factors. All the prescribed treatments will be recorded in a standardized electronic case report form (eCRF).

### Data collection and management

Eligible patients will be consecutively screened and included in the SUNRISE registry. All data will be collected via a real-time online data entry system with a predefined eCRF designed for this registry. Baseline characteristics including demographics, medical history, vital signs, the Glasgow Coma Scale (GCS) [23], the National Institutes of Health Stroke Scale (NIHSS) [24], the modified Rankin Scale (mRS) [25], the Barthel Index (BI) [26], Diagnostic Scale of Syndrome Elements in Ischemic Stroke (DSSEIS) [27], pictures of tongue, time of stroke onset, stroke subtypes, and laboratory test, will be collected accurately by investigators in each site. In addition, the details of the SHX treatments and the prescribing information of the treating physician will be documented. Several approaches will be used to minimize the potential heterogeneity or inaccuracy of data according to guidelines [28, 29]. Briefly, investigators from all sites will be trained before enrollment to understand the study protocol and will be required to comply with the standard operation procedure. Furthermore, an independent monitoring group will be responsible for reviewing the eCRFs for data quality. We will assign professionals to review data quality through a combination of online and on-site monitoring, including the timeliness, completeness, accuracy and authenticity of data records. Online data monitoring will be completed instantly, and we will require data revisions to be completed within seven days. Additionally, regular on-site monitoring will be conducted and monthly monitoring reports will be issued.

### Follow-up and study outcome

Local investigators at each site will conduct face-to-face or telephone follow-ups at 3 months (± 7 days), 6 months (± 10 days), and 12 months (± 14 days). An overall schedule of study-related visit and data collection of this registry is shown in Table 1. Briefly, data collection during the follow-up includes: (1) the mRS score will be assessed at 3, 6 and 12 months; (2) the Mini-Mental State Exam (MMSE) [30] will also be assessed at 3, 6 and 12 months; (3) the major events including death, ischemic stroke, intracerebral hemorrhage, myocardial infarction, heart failure and bleeding will be recorded during the follow-up period; (4) Adherence to the SHX treatment will be documented throughout the registry period.

**Table 1:** Schedule of study-related activities

	STUDY PERIOD				
	Baseline	Discharge	Follow-up		
Timepoint	T_0_	T_1_	T_2_ (3 months)	T_3_ (6 months)	T_4_ (12 months)
**Enrollment**					
Eligibility screen	**×**				
Informed consent	**×**				
Demographics	**×**				
Medical history	**×**				
Physical examination	**×**				
Neurological evaluation	**×**				
Previous medication	**×**				
CT/MRI	**×**				
ECG	**×**				
Laboratory examination	**×**				
Stroke subtypes		**×**			
Medications		**×**	**×**	**×**	**×**
**Assessments**					
NIHSS	**×**	**×**			
GCS	**×**	**×**			
mRS	**×**	**×**	**×**	**×**	**×**
BI	**×**	**×**			
MMSE			**×**	**×**	**×**
PRO		**×**			
TCM Syndrome	**×**	**×**			
Major vascular events		**×**	**×**	**×**	**×**
Overall mortality		**×**	**×**	**×**	**×**
Complications		**×**	**×**	**×**	**×**

**Abbreviations**: CT, Computed tomography scan; MRI, Magnetic resonance imaging; ECG, electrocardiogram; NIHSS, National Institutes of Health Stroke Scale; GCS, Glasgow Coma Scale; mRS, modified Rankin Scale; BI, Barthel Index; MMSE, Mini-Mental State Exam; PRO, Patient report outcome scale of stroke; TCM, Traditional Chinese Medicine

The primary outcome of the SUNRISE registry is the proportion of patients independent at 3 months after stroke onset defined by an mRS score of 0, 1, or 2. Secondary outcomes include the mRS score at 6 and 12 months; the change of NIHSS score from baseline to discharge; the BI score at discharge; the MMSE score at 3, 6, and 12 months; the GCS score at discharge, the proportion of patients reported major events of interest.

### Statistical analysis

The SUNRISE registry will be implemented in stages due to time and budget constraints. Aiming to initially gather data on acute ischemic stroke patients treated with SHX, describe and analyze the effects of SHX in real-world practice setting, and provide dependable data support for sample size calculation and protocol design in subsequent phases, the first phase is exploratory and has a target number of 1,000 participants. The next phases of this registry will be determined after the first phase of 1000 participants are completed. Baseline characteristics will be described using standard statistics. All baseline variables will be summarized using means and SDs for continuous variables that were normally distributed, while categorical variables were expressed as absolute frequencies and percentages. Given that the SUNRISE registry is one of the cohorts in the disease-specific CASES-TCM registry, a control group will be selected from the CASES-TCM population using a propensity score matching (PSM) approach to compare the effectiveness of SHX pill for acute ischemic stroke with those not receiving SHX treatment. The PSM will be performed with demographic and baseline characteristics. In each cohort, participants will be matched 1:1 based on propensity scores generated by logistic regression, using the nearest neighbor technique without replacement, with a maximum aperture of 0.2. After PSM, the comparability of outcomes between SUNRISE cohort and CASES-TCM cohort will be performed using a 2-sample t-test, one-way analysis of variance (ANOVA), or nonparametric tests (the $\:{\chi\:}^{2}$ test, Fisher’s exact test, or Mann-Whitney U test) as appropriate. Statistical analyses will be done using R software, version 4.1.2. Two-sided P values of less than 0.05 were accepted as statistically significant. A repeated measures analysis of variance (ANOVA) or mixed regression model will be used for repeated measurement data. To measure the relationships between multidimensional variables and outcomes of interest, multivariate regression analysis will be used in this study.

In addition, missing data are of great importance in real-world studies, in which missing data may cause seriously compromised inferences to scientific credibility of causal conclusion. Hence, we filled in the missing data using multiple imputations with predictive mean matching to ensure data integrity and comparability [31]. Moreover, in this real-world registry, potential confounding factors such as age, sex, smoking, body mass index, and severity of stroke, could be addressed in several ways [32]. The PSM approach will be used for the adjustment of measured confounders to ensure the balance between groups.

Pre-defined subgroup analyses will be performed of the effect of SHX at 3 months, subdivided by age, sex, severity of stroke, type of stroke, and TCM syndrome or TCM symptom.

## Discussion

In the era of evidence-based medicine, RCTs and systematic reviews are the most reliable approaches to determine the efficacy and safety of treatment [33]. Doctors and patients mainly take into account relevant findings from RCTs and systematic reviews in making treatment decisions. However, there is a growing interest in whether the findings from RCTs can be reasonably applied to patients in a real-world clinical practice setting [34]. The external validity (or generalizability) of RCTs is sometimes poor, which could be mainly attributed to relatively strict inclusion and exclusion criteria. Usually, in well-controlled settings, RCTs set strict inclusion and exclusion criteria to ensure the homogeneity of target population, and eliminate potential bias by using blinded allocation, placebo control methods. These procedures increase the internal validity, but will inevitably lead to underestimation of the effectiveness of treatment in real-world clinical practice. On the contrary, patients in routine practice settings may often be older, with more complications. Hence, the promising results from real-world studies are not always consistent with those from RCTs. The real-world evidence is becoming more and more important in clinical practice because it can offer more insightful data on the long-term effectiveness and safety of treatments for patients receiving treatments in real-world clinical settings [35–37].

The SUNRISE registry is a multicenter, prospective, product-specific, observational study, which was designed as one of the intervention cohorts in the China Stroke Registry for Patients with Traditional Chinese Medicine (CASES-TCM) [21]. The CASES-TCM registry is a large-scale, prospective, multicenter, disease-specific, observational study, attempting to depict major clinical characteristics of stroke in patients treated with TCM and to explore the effectiveness in the comparison with different TCM medications, with an anticipated sample size of 20,000. Besides the SUNRISE registry, there are several ongoing real-world registries belonging to the cohorts of the CASES-TCM registry.

There are several limitations in this study. First, a sample size of 1000 is relatively small for real-world registry. In the first phase of SUNRISE registry, 1000 patients will be enrolled initially, however, the subsequent phases of this registry will be determined after the first phase of 1000 participants are completed. Second, we only include patients with acute ischemic stroke, so we cannot investigate whether SHX is effective for intracerebral hemorrhage (ICH) in the current registry. In the subsequent phases of SUNRISE registry, both AIS and ICH patients will be included. And then, more findings could be established to understand the comprehensive benefits of SHX for acute stroke.

In addition to the primary analysis of the SUNRISE registry, several exploratory analyses could be done. First, the effectiveness and safety of SHX pill in the present real-world registry could be compared with the data from previous RCTs with PSM approach. Second, the characteristics of patients who present much greater response to SHX pill could be demonstrated in the current study, which will be undoubtedly helpful to guide the proper decision-making of SHX treatment, and enabling the personalized treatment. Third, the advantages of SHX pill for AIS could be summarized based on the data from the real-world setting, which may be helpful in the repositioning of SHX pill as a post-marketing drug. The findings of the SUNRISE registry will not only provide insights into the characteristics of patients who may benefit from SHX treatment, but also may enable the individualized treatment decision-making of SHX pill.

## Electronic supplementary material

Below is the link to the electronic supplementary material.

Supplementary Material 1: S1 File. Ethical review approval (English).

Supplementary Material 2: S2 File. Ethical review approval (Original Chinese).

Supplementary Material 3: S3 File. SPIRIT 2013 checklist.

## Data Availability

No datasets were generated or analysed during the current study. All relevant data for this study will be made available from the corresponding author on reasonable request upon study completion.
