# Supplementary material for: Suhexiang pill for acute ischemic stroke in real-world practice setting (SUNRISE): protocol of a multicenter registry
Source: BMC Complement Med Ther. 2025 Jan 28;25:30. doi: 10.1186/s12906-025-04762-9 (PMC11773706; doi:10.1186/s12906-025-04762-9)
Supplement: Supplementary file 1 — Supplementary Material 1: S1 File. Ethical review approval (English). [file 12906_2025_4762_MOESM1_ESM.pdf]

Ethics Committee of Dongzhimen Hospital affiliated to Beijing University of Chinese Medicine

Approval Notice Template

Approval number: 2022DZMEC-268-02

Study title: Suhexiang Pill for Acute Ischemic Stroke in Real-world Practice Setting: A Multicenter Registry

Application institute: Dongzhimen Hospital affiliated to Beijing University of Chinese Medicine

Principal investigator: Pro. Ying Gao, Xinxing Lai

Ethical review decision: Agree to this clinical study

Chairman of IRB: Pro. Hongfang Liu

Date of approval: September 29, 2011
