# Supplementary material for: Suhexiang pill for acute ischemic stroke in real-world practice setting (SUNRISE): protocol of a multicenter registry
Source: BMC Complement Med Ther. 2025 Jan 28;25:30. doi: 10.1186/s12906-025-04762-9 (PMC11773706; doi:10.1186/s12906-025-04762-9)
Supplement: Supplementary file 2 — Supplementary Material 2: S2 File. Ethical review approval (Original Chinese). [file 12906_2025_4762_MOESM2_ESM.pdf]

伦理审查批件

Approval Notice Template

项目受理号：2022DZMEC-268-02

项目名称：苏合香丸治疗缺血性中风临床疗效特点及安全性评价多中心注册登记研究	
主要研究者：高颖、赖新星	项目类别：科研课题
组长/参加：多中心，组长	
申办单位：北京中医药大学东直门医院	合同研究组织（CRO）：不适用
审查类别：复审	审查方式：■快速审查 □会议审查
审查委员：杜雅薇,赵波	审查日期：2022 年 09 月 28 日

批准文件：

1.修正的临床研究方案 （版本号：V2.0, 版本日期：2022 年 09 月 18 日）

2.修正的知情同意书 （版本号：V2.0, 版本日期：2022 年 09 月 18 日）

3.招募广告 （版本号：V1.0, 版本日期：2022 年 07 月 10 日）

4.病例报告 （版本号：V1.0, 版本日期：2022 年 07 月 10 日）

审查依据：根据 NMPA《药物临床试验质量管理规范》（2020 年）、《医疗器械临床试验质量管理规范》（2022 年）、世界医学会《赫尔辛基宣言》（2013 年）、国际医学科学组织委员会《涉及人的生物医学研究国际伦理指南》（2002 年）、国家卫生健康委员会《涉及人的生物医学研究伦理审查办法》（第 11 号，2016）等

审查决定：同意

注：

1、请按照伦理委员会规定的年度/定期跟踪审查频率 12 个月，申请人在截止日期前 1 个月提交研究进展报告。

2、本批件自签发日期有效期 12 个月，起止时间 2022-09-29 ~ 2023-09-28 ，研究者必须严格使用经审查同意的知情同意书和研究方案。

3、经伦理委员会批准的研究项目在实施前，研究项目负责人应当将该研究项目的主要内容、伦理审查决定在医学研究登记备案信息系统中进行登记备案。

4、凡涉及中国人类遗传资源、需要报批的研究项目，需告知在获得中国人类遗传资源管理办公室批准后才能开始。

5、研究过程中若变更主要研究者，对临床研究方案、知情同意书、招募材料等的任何修改，请申请人提

交修正案审查申请。

6、发生严重不良事件，请申请人及时提交严重不良事件报告。

7、研究纳入了不符合纳入标准或符合排除标准的受试者，符合中止试验而未让受试者退出研究。给予错误治疗或剂量，给予方案禁止的合并用药等没有遵从方案开展研究的情况；或可能对受试者的权益/健康以及研究的科学性造成不良影响等违背 GCP 原则的情况，请申办者/监查员/研究者提交违背方案报告。

8、申请人暂停或提前终止临床研究，请及时提交暂停/终止研究报告。

9、完成临床研究，请申请人提交结题报告。

主任委员☑副主任委员☐签字： 时 间：2022 年 09 月 29 日

北京中医药大学东直门医院医学伦理委员会

（盖章） 会议地点：无

本项目持续审查频率☐3 个月 ☐6 个月 ■12 个月 **联系人：韩雪婷 010-84012709**
